# Supplementary material for: Lower Respiratory Tract Infections in Pediatric Patients with Severe Neurological Impairments: Clinical Observations and Perspectives in a Palliative Care Unit
Source: Children (Basel). 2022 Jun 8;9(6):852. doi: 10.3390/children9060852 (PMC9221664; doi:10.3390/children9060852)
Supplement: Supplementary file 1 [file children-09-00852-s001.zip › children-1735900-supplementary.pdf]

children

MDPI

Table S1. Main and secondary diagnose.

Main diagnoses	*n* = 20
Hypoxic-ischemic encephalopathy	2 (10)
Complex developmental disorder of unclear origin	1 (5)
De-Lange Syndrome	1 (5)
Edwards syndrome	1 (5)
Epileptic encephalopathy	1 (5)
Epileptic encephalopathy (ARV1 mutation)	1 (5)
Epileptic encephalopathy (CACNA1E mutation)	1 (5)
Epileptic encephalopathy (SPTAN1 duplication)	1 (5)
Holoprosencephaly	1 (5)
Krabbe disease	1 (5)
Leukencephalopathy	1 (5)
Metachromatic leukodystrophy	1 (5)
Nemaline myopathy (ACTA 1 deficiency)	1 (5)
Neonatal cerebral leukomalacia following preterm birth	1 (5)
Nonketotic hyperglycinemia	1 (5)
Opitz trigonocephaly (C) syndrome	1 (5)
Perinatal asphyxia and hypoxic-ischemic encephalopathy	1 (5)
Pontocerebellar hypoplasia	1 (5)
Severe neurodevelopmental disorder of unknown origin	1 (5)
**Secondary diagnoses**	
Symptomatic focal epilepsy	10 (50)
Bilateral spastic cerebral palsy	8 (40)
Hypotonia	3 (15)
Bilateral spastic tetraparesis	3 (15)
Structural focal epilepsy	2 (10)
Obstructive sleep apnea	2 (10)
Symptomatic epilepsy	2 (10)
High-flow nasal canula	1 (5)
Bilateral hippocampal sclerosis	1 (5)
Dyskinesia	1 (5)
Epilepsy	1 (5)
Epilepsy and infantile spasms	1 (5)
Hypertrophic cardiomyopathy	1 (5)
Metabolic epilepsy	1 (5)
Chronic respiratory failure	1 (5)
Tracheostomy	1 (5)
Ventricular septal defect	1 (5)

Counts as *n* (% of included cases)
